# TarDB: an online database for plant miRNA targets and miRNA-triggered phased siRNAs

**DOI:** 10.1186/s12864-021-07680-5

**Published:** 2021-05-13

**Authors:** Jing Liu, Xiaonan Liu, Siju Zhang, Shanshan Liang, Weijiang Luan, Xuan Ma

**Affiliations:** College of Life Sciences, Tianjin Key Laboratory of Animal and Plant Resistance, Tianjin Normal University, Tianjin, 300387 China

**Keywords:** Plant, miRNA target, PhasiRNA, Degradome, Database

## Abstract

**Background:**

In plants, microRNAs (miRNAs) are pivotal regulators of plant development and stress responses. Different computational tools and web servers have been developed for plant miRNA target prediction; however, in silico prediction normally contains false positive results. In addition, many plant miRNA target prediction servers lack information for miRNA-triggered phased small interfering RNAs (phasiRNAs). Creating a comprehensive and relatively high-confidence plant miRNA target database is much needed.

**Results:**

Here, we report TarDB, an online database that collects three categories of relatively high-confidence plant miRNA targets: (i) cross-species conserved miRNA targets; (ii) degradome/PARE (Parallel Analysis of RNA Ends) sequencing supported miRNA targets; (iii) miRNA-triggered phasiRNA loci. TarDB provides a user-friendly interface that enables users to easily search, browse and retrieve miRNA targets and miRNA initiated phasiRNAs in a broad variety of plants. TarDB has a comprehensive collection of reliable plant miRNA targets containing previously unreported miRNA targets and miRNA-triggered phasiRNAs even in the well-studied model species. Most of these novel miRNA targets are relevant to lineage-specific or species-specific miRNAs. TarDB data is freely available at http://www.biosequencing.cn/TarDB.

**Conclusions:**

In summary, TarDB serves as a useful web resource for exploring relatively high-confidence miRNA targets and miRNA-triggered phasiRNAs in plants.

**Supplementary Information:**

The online version contains supplementary material available at 10.1186/s12864-021-07680-5.

## Background

In plants, microRNAs (miRNAs) are endogenous ~ 21 nucleotide (nt) non-coding RNAs, which are loaded into ARGONAUTE1 (AGO1) forming RNA-induced silencing complex (RISC) to direct RNA cleavage or translational repression of target transcripts [1–5]. Early studies well established that plant miRNAs pair with their target RNAs in a near-complementary manner [6], and demonstrated that plant miRNAs act through endonucleolytic cleavage of target RNAs [7, 8]. Meanwhile, emerging evidence suggests that translational repression is an important mode of miRNA actions in plants [9–11].

To fully understand miRNA-target RNA interactions, miRNA target prediction and validation become vital. Plant miRNA targets can be more readily predicted as compared with animal miRNA targets, due to the extensive complementarity between miRNAs and target RNAs [12, 13]. Bioinformatics tools or web servers such as Targetfinder, psRNATarget, psRobot, comTAR, TAPIR and TarHunter have been developed to predict miRNA targets in plants [14–19]. The detailed protocols of implementing these tools were recently reviewed [20].

All above plant miRNA target prediction programs are based on in silico analysis, while the rapid development of high throughput degradome/PARE (Parallel analysis of RNA ends) sequencing techniques have enabled to experimentally characterize miRNA cleavage sites at genome-wide scale. Accordingly, a few computational pipelines such as CleaveLand, PARESnip and sPARTA were developed to analyse degradome/PARE-seq datasets [21–23].

In addition, miRNA initiated *trans*-acting small interfering RNAs (tasiRNAs) or phased small interfering RNAs (phasiRNAs) have been implicated to play crucial roles in regulating plant growth and stress responses [24–26]. In Arabidopsis, phasiRNAs are predominantly 21-nt in length and are produced from limited numbers of gene loci including *TAS*, *PPR*, *AFB* and *NBS-LRR* [27, 28]. In other non-model plants, both 21-nt and 24-nt phasiRNAs were found; they are derived from hundreds to thousands of genomic loci, and a subset of them are particularly enriched in the reproductive tissues [29–38]. 22-nt miRNA has been recognized as a trigger for phasiRNA production [28, 39]. A “two-hit” model for miR390 triggered phasiRNAs at *TAS3* locus was well characterized, and miR390-*TAS3* interaction occurs in evolutionarily conserved manner [40]. By analysing polysome-bound small RNAs (sRNAs) in Arabidopsis, Li et al. showed that endoplasmic reticulum (ER) is an important site of phasiRNA initiation [41]. Recently, Yang et al. showed that miRNA-induced cleavage occurs on ER-bound polysomes in maize and rice [42].

Given the essential regulatory roles of miRNAs and phasiRNAs, it is highly necessary to systematically integrate miRNA target prediction, degradome/PARE-seq analysis and miRNA-triggered phasiRNA identification to create a high-confidence miRNA target database in plants. Currently, a few plant miRNA databases such as miRBase [43] and PmiREN [44] have been established; PmiREN also contains miRNA target data. PmiREN extensively focuses on miRNAs, while the miRNA target data on PmiREN are incomplete; for example, *Oryza sativa* miR2118 has over 1000 target sites in the genome, whereas PmiREN collects very limited numbers of miR2118 targets. Several plant miRNA target prediction web servers such as psRNATarget [17], psRobot [16] and WPMIAS [45] have been reported, but they lack miRNA-initiated phasiRNA information. The pipelines *PHASIS* (https://github.com/atulkakrana/PHASIS) and PhaseTank [46] were developed to predict phasiRNAs in plants. Recently, Chen et al. developed sRNAanno, a database that has comprehensive collection of phasiRNA loci in plants [47]. sRNAanno does not indicate which phasiRNA sites are triggered by miRNAs.

To this end, we have systematically analysed plant miRNA targets and miRNA-triggered phasiRNAs, and constructed TarDB database, which collects 62,888 cross-species conserved miRNA targets, 4304 degradome/PARE-seq supported miRNA targets and 3182 miRNA-triggered phasiRNA loci. TarDB collects high-confidence miRNA targets and serves as a useful resource for future studies in plant sRNA field.

## Construction and content

### Data resource

The degradome/PARE-seq data used to create TarDB were downloaded from NCBI GEO or SRA databases (http://www.ncbi.nlm.nih.gov). For some raw sequencing data, the adaptor sequences were detected by FastQC (http://www.bioinformatics.babraham.ac.uk/projects/fastqc/), and then were trimmed using Cutadapt (https://cutadapt.readthedocs.io/en/stable/). The sRNA-seq data were retrieved from NCBI GEO or Donald Danforth Plant Science Center (http://smallrna.danforthcenter.org/) or Dr. Blake Meyers’s lab website (https://mpss.meyerslab.org/). Plant genomic and transcript sequences as well as annotations were derived from JGI Phytozome (https://phytozome.jgi.doe.gov/). Gene ontology terms for each transcript were downloaded from Phytozome BioMart (version 12). The mature and precursor miRNA sequences were derived from miRBase (http://www.mirbase.org/) or PmiREN (http://www.pmiren.com/) or Plant sRNA Gene Sever at Pennsylvania State University (https://plantsmallrnagenes.science.psu.edu/). The secondary structures of precursor miRNAs were generated using Perl module RNA::HairpinFigure (https://metacpan.org/pod/RNA::HairpinFigure). The graphs of different plant species were downloaded from www.plantgenera.org. The details of the data resources used for constructing TarDB are included in Supplementary Table S1.

### Analysis procedure

Our workflow of creating TarDB is depicted on the “Guide” page (http://www.biosequencing.cn/TarDB/guide/guide.html), which includes three parts. In part I, the cross-species conserved miRNA targets were identified using TarHunter [18] with homo mode and score ≤ 5. The homo mode requires the 50-nt upstream and downstream regions of miRNA target sites are cross-species conserved. Then, the results were parsed by in-house Perl scripts to generate the webpages in HTML format. In part II, The degradome/PARE-seq supported targets were identified by CleaveLand4 [21] with category ≤2, Allen et al. score [12, 14] ≤5 and *P*-value ≤0.05. The degradome signature plots in PDF format were converted to PNG format using ImageMagick (https://imagemagick.org/index.php) with density of 100. In part III, the phasiRNA loci were identified following previously well-documented approach [27, 28, 30, 31, 48] with minor modifications. Briefly, the processed sRNA reads were first mapped to genome using ShortStack (https://github.com/MikeAxtell/ShortStack) allowing no mismatch, and the assignment of multi-mapping reads was guided by unique mapping reads (option --mmap u). The key parameters for executing ShortStack is as follows: --bowtie_m 100 --ranmax 50 --mmap u --mismatches 0 --nostitch. Next, the sRNA reads from genomic Watson and Crick strands were unified and the phasing scores were calculated as previously described [48]. Subsequently, the hypergeometric test (*P*-value < 0.01) was performed to obtain candidate phasiRNA loci [28, 49]. PhasiRNA analysis algorithms and scripts have been reported previously, such as *PHASIS* (https://github.com/atulkakrana/PHASIS) and PhaseTank [46]. We implemented TarHunterL [18] to predict possible miRNA target sites at each phasiRNA locus, and then retrieved the loci with predicted miRNA slicing site locating at phasing positions. We performed the above steps using in-house Perl and R scripts, which enabled to automatically generate the graphs of sRNA reads profiles and phasing score plots at different phasiRNA loci. Finally, we manually inspected the graph of each phasiRNA locus to guarantee the phasing quality.

### Database construction

TarDB database was placed on a web server with Linux CentOS6.2 operating system. The webpages at TarDB were created using HTML (Hypertext Markup Language) and CSS (Cascading Style Sheets), and were rendered by Bootstrap version 4.4 (https://getbootstrap.com/), Layui (https://www.layui.com/) and jQuery (https://jquery.com/). Several plugins were downloaded for interactive displaying, such as jsTree (https://www.jstree.com/) for showing interactive tree. TarDB database was managed by MySQL (https://www.mysql.com). PHP (pre hypertext processor, version 5.6) scripts were implemented at server end for querying MySQL database.

## Utility and discussion

### Database details

**Fig. 1:**
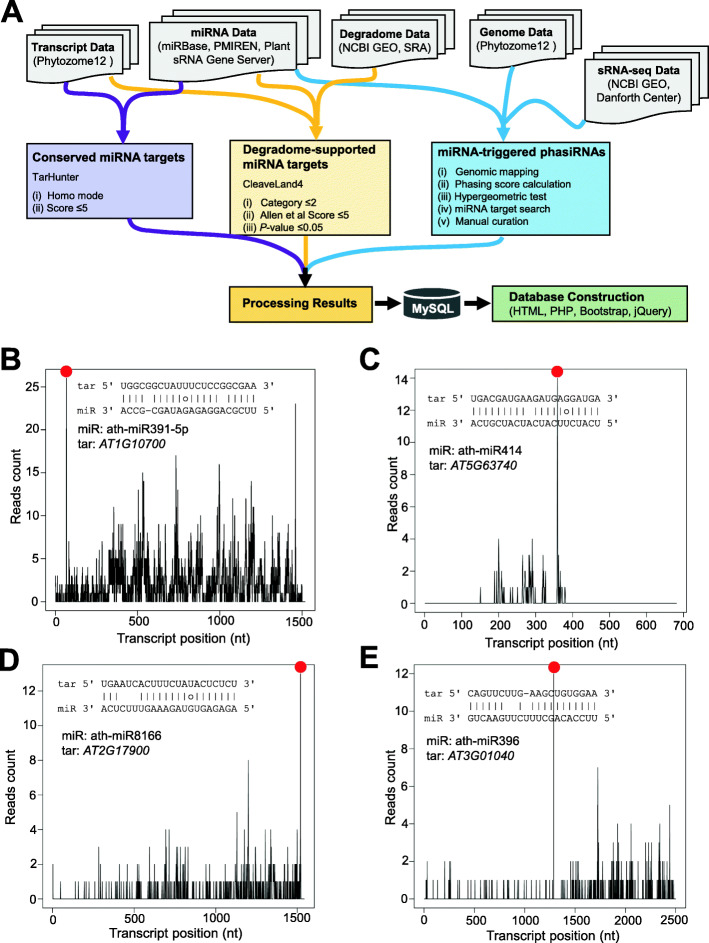
Workflow of TarDB construction and examples of new miRNA targets in Arabidopsis. **a** Procedure of sequencing data analysis and database construction. TarDB contains three sections including conserved miRNA targets (left), degradome-supported miRNA targets (middle) and miRNA-triggered phasiRNAs (right). The key parameters used in each analysis are shown. **b**, **c**, **d** and **e** are new miRNA targets supported by degradome/PARE-seq in the model species *Arabidopsis thaliana*. miRNA-target pairing is shown within the degradome signature plot. miRNA induced cleavage site is marked by a red dot

Our workflow of constructing TarDB database is depicted in Fig. 1a. The miRNA target data deposited at TarDB consist of three categories: cross-species conserved miRNA targets, degradome/PARE-seq supported miRNA targets and miRNA-triggered phasiRNAs.

The conserved miRNA targets were identified by TarHunter, our previously reported tool that is based on the rational that homologous miRNAs target homologous sequences among diverse species [18]. TarDB collects a total of 62,888 conserved miRNA targets with cutoff score of 5, which fall into 4775 conserved groups from 43 plant species. These species range from green algae to higher flowering plants, including 24 dicotyledonous and 12 monocotyledonous plants, 1 basal angiosperm, 1 gymnosperm, 3 bryophytes and 2 algae species. The phylogenetic relationships of these 43 species are shown in Additional file 1: Supplementary Fig. S1. Without conservation filter, TarHunter identified 539,420 miRNA-target pairs; thus, the conservation filter greatly narrows down the target gene list and increases the prediction confidence. It is worth noting that TarHunter analysis is based on *in silico* prediction of cross-species conserved miRNA target sites, and may produce false positive results. If users aim to obtain highly reliable miRNA-targeted transcripts, they can choose the degradome/PARE-seq option on TarDB.

The degradome/PARE-seq analysis was based on Phytozome annotated transcript database. Degradome/PARE-seq supported miRNA targets were identified by CleaveLand4 [21] with score ≤ 5 and *P*-value ≤0.05. Only the data belonging to degradome categories 0, 1 and 2 data are displayed on TarDB, since these categories represent relatively reliable cleavage sites. Degradome/PARE-seq has been the most effective and high-throughput approach for capturing miRNA target sites at genome-wide scale in plants. Through analysis of 51 published degradome/PARE-seq datasets (Additional file 2: Supplementary Table S1), we obtained a total of 4304 degradome-supported high-confidence miRNA targets from 18 plants. TarDB collects novel degradome-supported miRNA targets even in the well-studied model species. Take *Arabidopsis thaliana* as an example: we identified 233 miRNA-target pairs (gene isoforms were counted once) in *A. thaliana* using the following criteria: (i) category 0 or 1; (ii) score ≤ 5; (iii) *P*-value ≤0.01. The majority of these miRNA-target interactions have been characterized previously, but there remains a handful of potential new miRNA targets that need further investigations as shown below. In Arabidopsis, miR391 targets *PRS3* (*AT1G10700*), a P-independent phosphoribosyl pyrophosphate (PRPP) synthase gene (Fig. 1b); miR414 targets *AT5G63740*, a gene encoding RING/U-box superfamily protein (Fig. 1c); miR8166 targets *ASHR3* (*AT2G17900*) that confers histone H3 lysine-36 methylation (Fig. 1d); miR396 regulates *AT3G01040* encoding a putative galacturonosyltransferase (Fig. 1e). In addition to model species, TarDB also collects many novel degradome/PARE-seq supported miRNA targets in diverse non-model species, a few of which will be mentioned in the “Case study” section.

The miRNA-triggered phasiRNA loci were identified following previously well-documented criteria [28, 30, 31, 48] and by manual curation. Currently, many plant miRNA target prediction tools or servers (e.g., Targetfinder, psRNATarget, psRobot) lack phasiRNA analysis function. Therefore, we incorporated phasiRNA data on TarDB platform allowing users to conveniently query miRNA-triggered phasiRNAs in plants. Through analysis of 176 published sRNA-seq datasets, we obtained 2275 21-nt and 338 24-nt miRNA-triggered phasiRNA loci from 21 species, and most of the phasiRNA triggering miRNAs are lineage specific (Additional file 1: Supplementary Fig. S1). Note that we identified a large numbers of phasiRNA candidate loci, but miRNA-triggered phasiRNAs only represent a small portion. Additionally, we discarded the phasiRNA loci with the predicted miRNA cleavage site not locating at phasiRNA register positions.

### Database interface

TarDB web database has six main interfaces including “Home”, “Browse”, “Search”, “Download”, “Guide” and “Contact”. The “Home” interface presents an overview of TarDB database. It contains an introduction of miRNA target regulations, and briefly describes the prior studies on conserved miRNA targets, degradome/PARE-seq technique and miRNA-triggered phasiRNAs in plants. It also consists of the basic statistics of TarDB data.

**Fig. 2:**
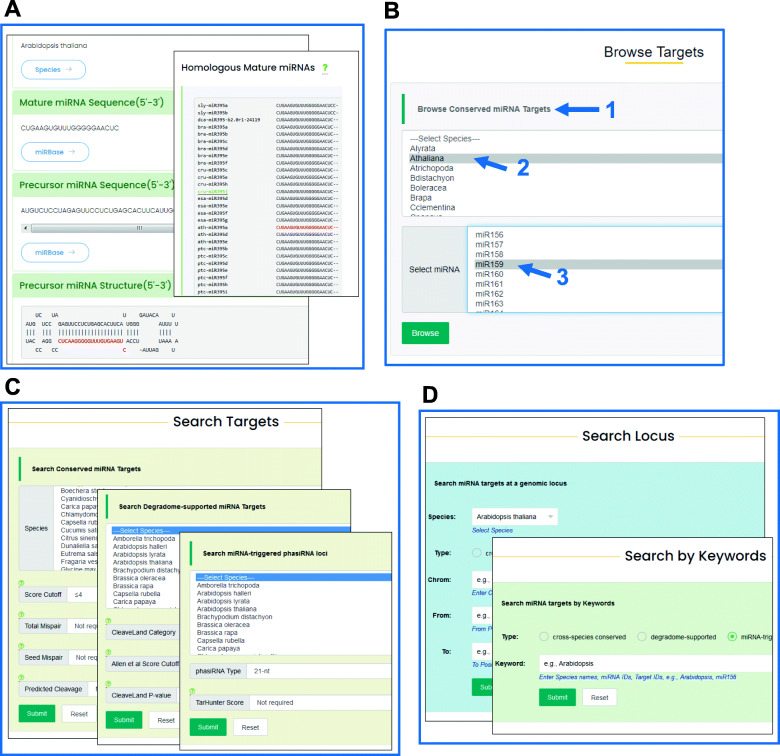
Screenshots of “miRNA”, “Browse” and “Search” pages on TarDB. (**a**) “miRNA” page includes sequence and structure information for mature and precursor miRNAs. It also displays the alignment of homologous miRNAs in related species. (**b**) The “Browse Targets” function on the “Browse” page enables users to obtain miRNA targets in a three-step way. (**c**) “Search” page allows users to search conserved miRNA targets, degradome/PARE-seq supported targets and miRNA-triggered phasiRNAs. (**d**) “Search” page allows users to search miRNA target gene(s) at a specific genomic locus or by using key words

The “Browse” interface allows users to browse various miRNA families, diverse plant species and the three types of miRNA targets on TarDB. The miRNA sequence data are mostly derived from miRBase (release 22) [43]. Some miRNA data are from PmiREN [44] and Plant sRNA Gene server [50]. Users can view the sequences and secondary structures of mature/precursor miRNAs, and click on the corresponding external links to obtain more miRNA information (Fig. 2a). The “Browse Targets” section offers users an easy three-step way to browse any miRNA target data on TarDB (Fig. 2b). First, users need to choose miRNA target type, and then select a species which will automatically generates a miRNA list. Finally, users can click a specific miRNA on the list to get access to relevant miRNA target data.

**Fig. 3:**
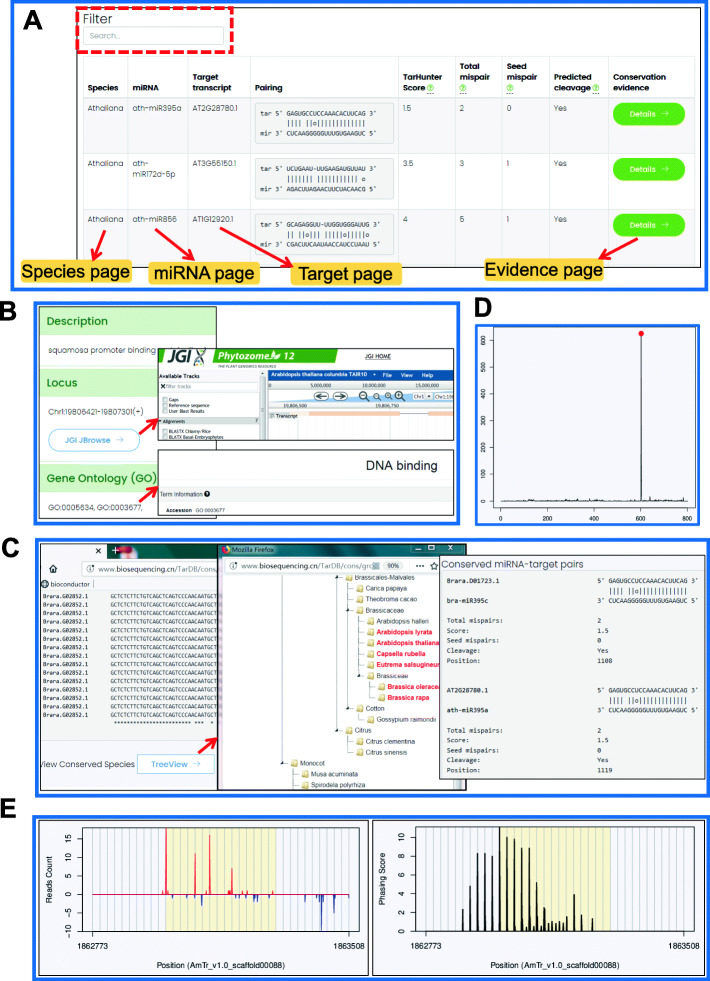
Screenshots of searching results and hyperlinked pages. **a** Searching results are shown in tabular format. Red dashed-line box indicates filtering function. Querying results can be further linked to species, miRNA, target and evidence pages. **b** “Target” page has hyperlinks to Phytozome Genome Browser and contains the information of GO identifiers. **c** Alignment of conserved miRNA target sites. Clicking the “Treeview” button displays the species having conserved miRNA targets. **d** Screenshot of degradome signature plot. **e** Screenshot of sRNA-seq reads (left) and phasing score (right) profiles. The reads mapping signals at genomic Watson and Crick strands are shown in red and blue colors, respectively. 21/24-nt intervals are marked by grey lines in phasing score plot

The “Search” interface is the key section of TarDB, and it comprises three modes. In the “Search target” mode, users can search conserved miRNA targets, degradome/PARE-seq supported miRNA targets and miRNA-triggered phasiRNAs with customizable parameters such as penalty scores, maximum mispairs, degradome category, *P*-value cutoff and phasiRNA types (Fig. 2c). In the “Search locus” mode, users can query different types of miRNA targets at a specific genomic locus in a specified species (Fig. 2d). In the “keyword search” mode, users can search miRNA targets by entering a keyword, e.g., species name, miRNA or transcript IDs (Fig. 2d). The searching results are displayed in tabular format (Fig. 3a). The results can be further narrowed down using a filtering box (red dashed-line box in Fig. 3a). Each resultant record has hyperlinks that navigate to specific species, miRNA, target and evidence webpages (red arrows in Fig. 3a). The “Target” page contains transcript sequence, functional annotation and Gene Ontology (GO) information (Fig. 3b). Users can also get access to JGI Phytozome transcript website or JGI genome browser to visualize gene structure in genomic content (Fig. 3b). The “Evidence” page presents detailed supporting information for certain miRNA-target regulations. For conserved miRNA targets, miRNA-target pairing patterns and sequence alignment of homologous target sites from various species are displayed (Fig. 3c). For miRNA targets with degradome/PARE-seq evidence, the Allen et al. score [12, 14], CleaveLand4 *P*-value and the degradome signature plot highlighting miRNA cleavage position are shown (Fig. 3d). For phasiRNA loci, the sRNA-seq reads profile and phasing score plot are displayed (Fig. 3e). Within the transcript sequence, the miRNA target site is marked in red color.

The “Download” interface shows a phylogenic tree of various plant species. Clicking on each species node allows users to download the corresponding miRNA target data as a zip compressed file. The “Guide” interface presents our workflow of sequencing data manipulation and database construction, as well as a step-to-step guidance for exploring the key features of TarDB. The “Frequently Asked Questions (FAQs)” section on the “Guide” page provides explanations for the parameters in searching different types of miRNA targets. The “Guide” page also contains the hyperlinks that navigate to related miRNA target web resources.

### Case study

Next, we present four case studies to illustrate the process of mining TarDB for identifying novel conserved miRNA targets, degradome-supported miRNA targets and miRNA-triggered phasiRNAs in plants.

#### Case I

In the “Search Conserved miRNA Targets” section on the “Search” page, users can query conserved miRNA targets using a combination of parameters. The default score cutoff is set to 4. Smaller scores indicate more stringent miRNA-target complementarities. Users can set total mispair cutoff value, i.e., total mismatches and Indels (insertions and deletions). Users can also adjust seed mispair cutoff value, i.e., total mispairs at miRNA 5′ positions 2–7. miRNA seed region is crucial for miRNA-target interaction in animals and plants [51, 52]; thus, we included this parameter in miRNA target search. The “predicted cleavage” is based on the previous observation that perfect match at miRNA 5′ positions 9–11 is crucial for miRNA-mediated cleavage [53].

Actually, TarDB provides flexible ways for searching conserved miRNA targets. We take miR391 as an example. We have mentioned above that Arabidopsis miR391 targets a PRPP synthase gene *AT1G10700* by means of degradome/PARE-seq analysis (Fig. 1b). To view miR391-*AT1G10700* interaction in phylogenic way, users can simply enter “miR391” in the keyword box on the “Search” page and select “cross-species conserved” target type, and then all conserved miR391 targets among different species will be displayed (Additional file 1: Supplementary Fig. S2A). Users can choose “*AT1G10700*” record to view its details (red circle in Additional file 1: Supplementary Fig. S2A). Clearly, the regulation between miR391 and PRPP synthase gene is conserved in four *Brassicaceae* species including *Arabidopsis thaliana*, *Arabidopsis lyrata*, *Capsella rubella* and *Brassica rapa* (Additional file 1: Supplementary Fig. S2B). Collectively, we can deduce that miR391 regulates PRPP synthase gene, which, to the best of our knowledge, has not been reported yet.

#### Case II

**Fig. 4:**
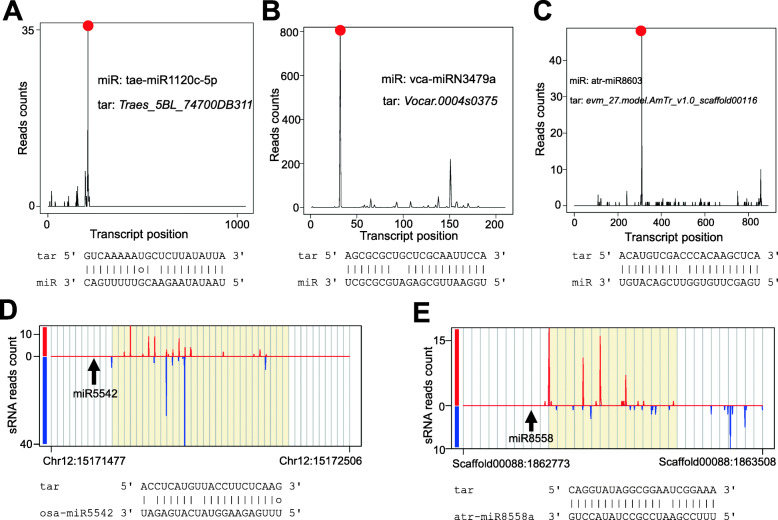
TarDB deposits novel miRNA targets and miRNA-triggered phasiRNA sites. **a** In wheat, tae-miR1120c-5p targets the gene *Traes_2DS_E6EDAED7B*, which is supported by degradome/PARE-seq analysis (data accession number: GSM911924). miRNA-target pairing is shown below the degradome signature plot. **b** In green alga *Volvox carteri*, miRN3479a cleaves an unknown transcript (data Acc. No.: GSM1263779). Gene ID is derived from Phytozome. **c** In *Amborella trichopoda*, miR8603 targets a gene encoding POZ/BTB domain protein (data Acc. No.: GSM1024603). Gene ID is derived from Phytozome. **d** *Oryza sativa* miR5542 triggers phasiRNAs evidenced by sRNA-seq analysis (data Acc. No.: GSM816732). sRNA-seq reads 5′ positions are plotted and miR5542 slicing site is indicated by an arrow. The red and blue lines indicate genomic Watson and Crick strands, respectively. The grey lines mark 21-nt intervals. **e** *A. trichopoda* miR8558 is able to trigger phasiRNAs with its cleavage site indicated by an arrow (data Acc. No.: GSM712477)

Degradome/PARE-seq provides a robust experimental evidence for miRNA directed cleavage of target RNAs in plants [54, 55]. One of the functionalities of TarDB is to search degradome/PARE-seq supported miRNA targets in various plants especially for non-model species. Take bread wheat, an important global cereal, as an example: in the “Degradome supported miRNA target” section on the “Search” page, users can choose “*Triticum aestivum*” from the species selection box, and then simply click the “Submit” button. This returns a list of 122 wheat miRNA-target pairs with degradome/PARE-seq evidence. Normally, our default settings are sufficiently strict to identify relatively high-confidence miRNA targets. Users can adjust appropriate parameters such as increasing Allen et al. score which identifies miRNA targets with relaxed pairing. Users can also select “Category 2”, which still identifies statistically significant degradome peaks but at the risk of getting false positives. Although wheat miRNA targets have been well reported [56–58], TarDB contains novel unreported wheat miRNA targets; for instance, miR1120 regulates a gene (*Traes_2DS_E6EDAED7B*) encoding peroxidase superfamily protein in wheat (Fig. 4a). Through mining TarDB, we could identify previously undocumented miRNA targets particularly in non-model species; for examples, miRN3479a cleaves an unknown transcript in the multicellular alga *Volvox carteri* (Fig. 4b), and miR8603 targets a gene encoding POZ/BTB domain protein in the ancient angiosperm species *Amborella trichopoda* (Fig. 4c).

#### Case III

tasiRNAs/phasiRNAs belong to one of the classic types of siRNAs found in plants [25, 59]. Although many phasiRNA triggers are unknown, compelling evidence suggests that 22-nt miRNAs are capable of triggering phasiRNA biogenesis [39]. In the “two-hit” model, 21-nt miRNAs such as miR161 and miR400 are also involved in phasiRNA production [40]. The rapid increase of sRNA-seq data facilitates identification of miRNA-initiated phasiRNAs in diverse plant species. Take miR2118, which triggers 21-nt phasiRNA production in a wide spectrum of angiosperms [25], as an example. A recent study in rice (*Oryza sativa*) reveals that miR2118-dependent phasiRNAs in the anther wall are characteristic of abundant U, and are essential for anther wall development [60]. To browse miR2118-triggered phasiRNAs in rice, users can simply enter “miR2118” in the keyword box on the “Search” page, and then filter the results by entering “Osativa”, which outputs 1112 candidate miR2118-triggered phasiRNA loci in rice genome. Apart from the known phasiRNA triggers, TarDB collects new triggers; for instance, *O. sativa* specific miR5542 is able to trigger phasiRNAs with its cleavage site locating exactly at phasing position (Fig. 4d). Take *Amborella trichopoda*, which represents a basal lineage of flowering plants, as another example. To probe miRNA-triggered phasiRNAs in this species, users can choose the species “*Amborella trichopoda”* and select a phasiRNA type (21-nt or 24-nt), and then click the “Submit” button. This identifies 27 21-nt phasiRNA loci in *A. trichopoda*. Interestingly, 11 of them are triggered by miR8558, a 22-nt miRNA (Fig. 4e). Users can click “miR8558” to get the details of this miRNA; evidently, it is specific in *A. trichopoda* and has no homologs in other species. In plants, miR8558-triggered phasiRNAs has not been reported; thus, TarDB provides a platform for mining novel miRNA-triggered phasiRNAs in plants.

#### Case IV

**Fig. 5:**
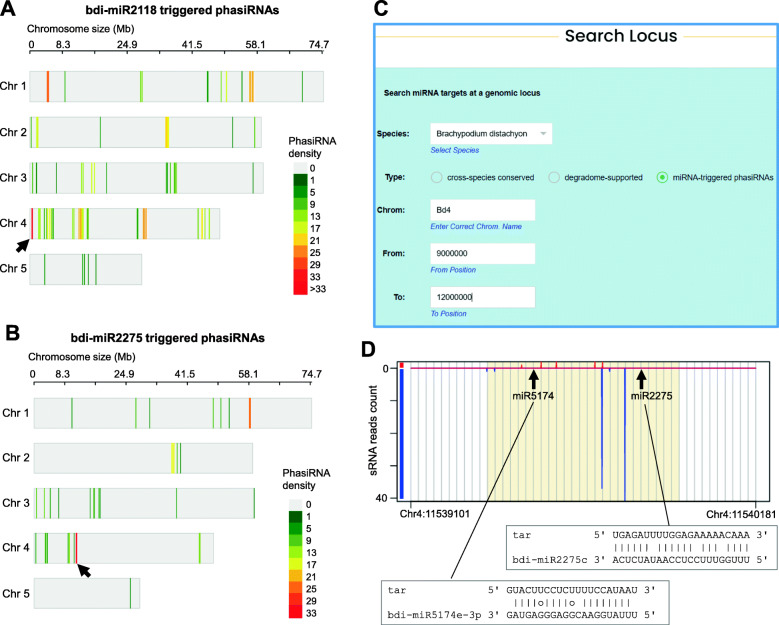
PhasiRNAs are enriched on chromosome 4. **a**, **b** Genomic distributions of miR2118 and miR2275 triggered phasiRNA loci in *Brachypodium distachyon*. The number of phasiRNA loci (phasiRNA density) is represented in color scale. Arrows indicated phasiRNA enriched genomic regions. **c** Screenshot of “Search Locus” interface. **d** The phasiRNAs produced at the locus Chr4:11539101–11,540,181(−) are likely to be triggered by miR2275 and miR5174 in “two-hit” mode. Genomic Watson and Crick strands are shown in red and blue colors, respectively. The grey lines in phasing score plot mark the 24-nt intervals

It has been well studied that in grass species, miR2118 and miR2275 trigger 21-nt and 24-nt phasiRNAs, respectively, during plant reproductive stage [33, 35]. Recently, Tian et al. reports evolutionary analysis of miR2118 and miR2275 triggered phasiRNAs in five *Oryza* species, and their study strengthens the viewpoint that phasiRNAs are able to cleave phasiRNA precursor in *cis* manner [61]. We examined miR2118 and miR2275 triggered phasiRNAs in *Brachypodium distachyon*, a cereal grain species closely related to the Triticeae crops. TarDB collects 499 miR2118 triggered phasiRNA loci and 194 miR2275 triggered phasiRNA loci in *B. distachyon*. Analysis of the genomic distributions of these phasiRNA loci shows that they are enriched on Chromosome 4 (Fig. 5a,b). Notably, both miR2118 and miR2275 triggered phasiRNAs are abundantly produced from two distinct regions on Chromosome 4 (indicated by arrows in Fig. 5a,b), which may be similar to the phasiRNA supercluster found in rice. TarDB has a “Search locus” function on the “Search” page, allowing users to search miRNA targets in specific genomic regions. For instance, with this function, users can query the phasiRNAs at positions 9–12 Mb on Chromosome 4 in *B. distachyon* (Fig. 5c). This returns a full list of 21-nt and 24-nt phasiRNA loci; 12 of them are triggered by miR2118, 4 are triggered by miR5163, and 51 are triggered by miR2275. Hence, miR2275-initiated phasiRNA loci are remarkably enriched at this 3 Mb region on Chromosome 4. Among them, the locus Chr4:11539101–11,540,181(−) may produce a transcript cleaved by miR2275 and miR5174 to induce 24-nt phasiRNAs in “two-hit” manner, as shown in Fig. 5d.

Taken together, TarDB will be useful for plant biologists who seek for high-confidence miRNA target sites. TarDB collects the targets of a comprehensive list of plant miRNAs, including both highly conserved and less conserved miRNAs. According to the data deposited on TarDB, we compiled the phylogenetic distributions of different miRNA families in plants, and labeled the miRNAs that have degradome/PARE-seq supported targets or are able to trigger phasiRNAs (Additional file 1: Supplementary Fig. S1). Many previous efforts were devoted to characterizing highly conserved miRNAs and their targets in plants; in recent years, the lineage-specific miRNAs have been attracting more attentions. On TarDB, the great majority of lineage-specific miRNAs have either degradome or phasiRNA evidence (Additional file 1: Supplementary Fig. S1), indicating lineage-specific miRNAs are evolutionarily functional and may need future investigations. TarDB also collects the targets for a large number of species-specific miRNAs having degradome-seq or sRNA-seq supported targets, which are not shown in Additional file 1: Supplementary Fig. S1. These species-specific miRNAs have proved to play pivotal roles in plant development and stress responses [5, 62, 63], and TarDB offers a platform to search their targets.

During degradome/PARE-seq analysis, we found many datasets of non-model species have limited sequencing depth; comparatively, the model species *A. thaliana* has numerous degradome/PARE-seq data derived from various tissues and treatments. Therefore, with the rapid development of NGS (next-generation sequencing) technologies, sequencing-based efforts toward non-model plants are highly needed. Acquisition of the degradomes/small RNA-omes from a great diversity of non-model species will provide further insights into miRNA-target co-evolution in plants. With the increasing genomic and sequencing data, we will update TarDB regularly and include more plant species and miRNA target data in the future.

## Conclusions

Here, we introduce TarDB, a miRNA target and miRNA-triggered phasiRNA database, which implements cross-species conservation and experimental filters to obtain relatively reliable miRNA targets. TarDB provides rich information and serves as a useful web resource for exploring high-confidence miRNA targets in plants. TarDB can be freely accessed at http://www.biosequencing.cn/TarDB.

## Supplementary Information

**Additional file 1: Supplementary Fig. S1.** Phylogenetic overview of plant miRNA targets with degradome or phasiRNA support. Phylogenetic relationship of 43 plant species on TarDB. Different plant groups are shaded in different colors. Plant miRNAs are generally divided into highly conserved and lineage-specific miRNA families; those species-specific miRNAs are not shown. According to TarDB data, the miRNAs that have degradome/PARE-seq supported targets are underlined in red, and the 22-nt miRNAs that trigger phasiRNAs are underlined in blue. **Supplementary Fig. S2.** The regulation between miR391 and PRPP synthase gene is conserved in *Brassicaceae* species. **(A)** Screenshot of miR391 target searching result. Red dashed line circle indicates the link to view the details of miR391-*AT1G10700* regulation. **(B)** The regulation between miR391 and PRPP synthase gene is conserved in four *Brassicaceae* species that are highlighted in red colors. Sequence alignments of different miR391 target sites are shown.

**Additional file 2: Supplementary Table S1.** Degradome/PARE-seq and small RNA-seq data used for TarDB database construction.

## Data Availability

TarDB can be freely accessed at http://www.biosequencing.cn/TarDB.

## Abbreviations

sRNA: Small RNA
miRNA: microRNA
phasiRNA: Phased small interfering RNA
tasiRNA: Trans-acting siRNA
AGO: ARGONAUTE
RISC: RNA-Induced silencing complex
PARE: Parallel analysis of RNA ends
ER: Endoplasmic reticulum
PRPP: Phosphoribosyl pyrophosphate
