# Supplementary material for: TarDB: an online database for plant miRNA targets and miRNA-triggered phased siRNAs
Source: BMC Genomics. 2021 May 13;22:348. doi: 10.1186/s12864-021-07680-5 (PMC8120726; doi:10.1186/s12864-021-07680-5)
Supplement: Supplementary file 1 — Additional file 1: Supplementary Fig. S1. Phylogenetic overview of plant miRNA targets with degradome or phasiRNA support. Phylogenetic relationship of 43 plant species on TarDB. Different plant groups are shaded in different colors. Plant miRNAs are generally divided into highly conserved and lineage-specific miRNA families; those species-specific miRNAs are not shown. According to TarDB data, the miRNAs that have degradome/PARE-seq supported targets are underlined in red, and the 22-nt miRNAs that trigger phasiRNAs are underlined in blue. Supplementary Fig. S2. The regulation between miR391 and PRPP synthase gene is conserved in Brassicaceae species. (A) Screenshot of miR391 target searching result. Red dashed line circle indicates the link to view the details of miR391-AT1G10700 regulation. (B) The regulation between miR391 and PRPP synthase gene is conserved in four Brassicaceae species that are highlighted in red colors. Sequence alignments of different miR391 target sites are shown. [file 12864_2021_7680_MOESM1_ESM.pdf]

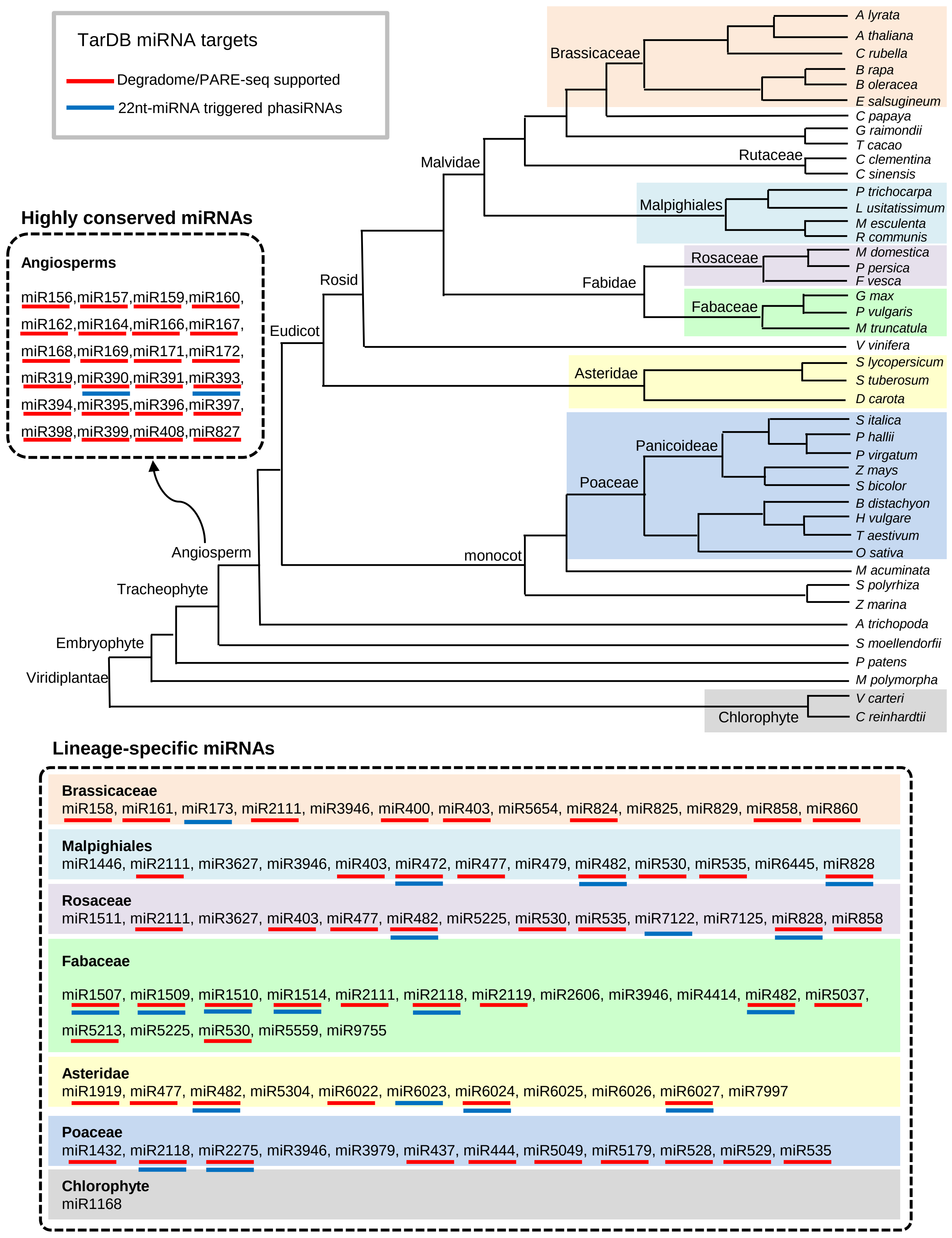

Supplementary Fig. S1 Phylogenetic overview of plant miRNA targets with degradome or phasiRNA support. Phylogenetic relationship of 43 plant species on TarDB. Different plant groups are shaded in different colors. Plant miRNAs are generally divided into highly conserved and less conserved miRNA families; those species-specific miRNAs are not shown. According to TarDB data, the miRNAs that have degradome/PARE-seq supported targets are underlined in red, and the 22-nt miRNAs that trigger phasiRNAs are underlined in blue.

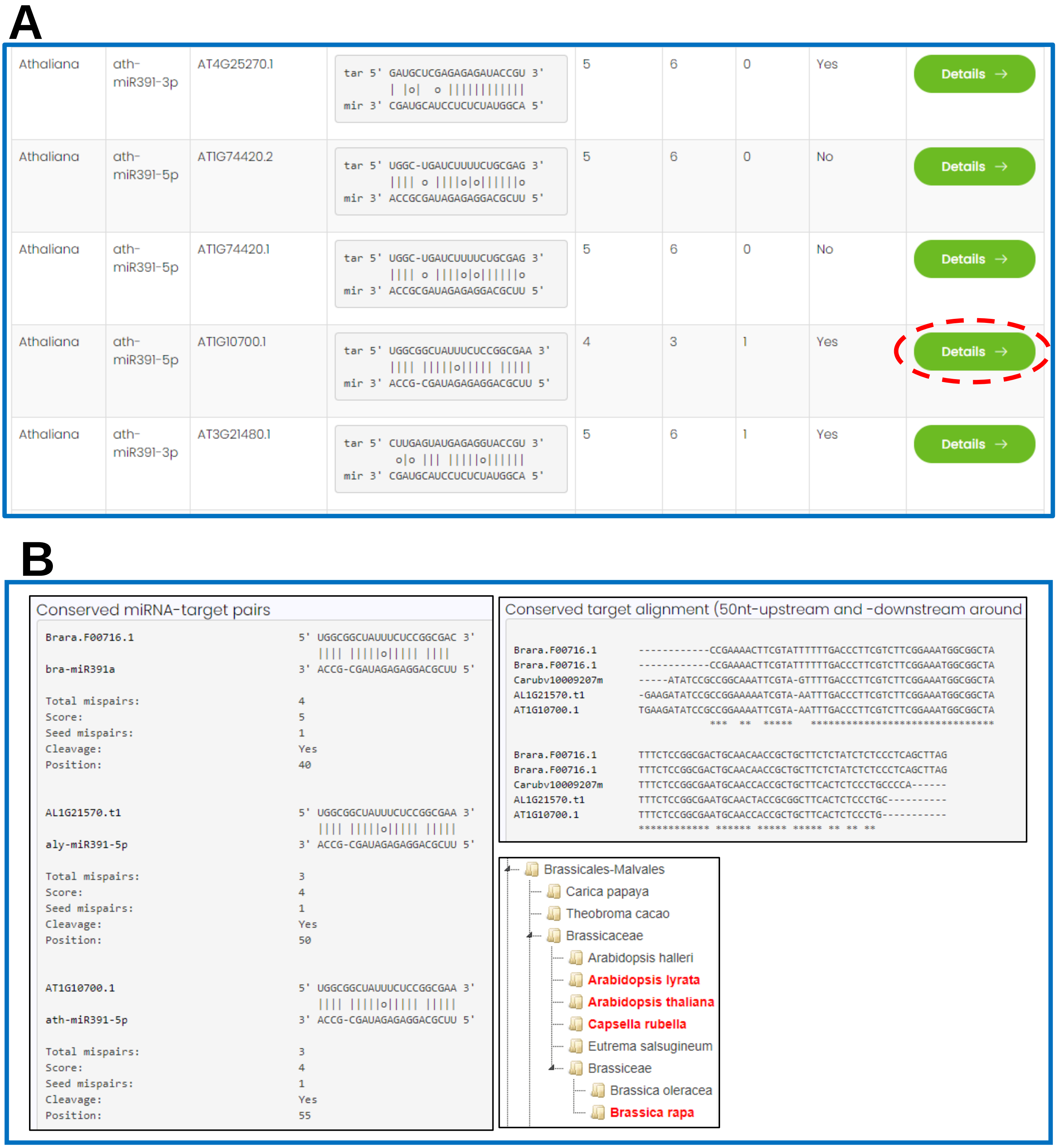

Supplementary Fig. S2 The regulation between miR391 and PRPP synthase gene is conserved in *Brassicaceae* species.

(A) Screenshot of miR391 target searching result. Red dashed line circle indicates the link to view the details of miR391-*AT1G10700* regulation. **(B)** The regulation between miR391 and PRPP synthase gene is conserved in four *Brassicaceae* species that are highlighted in red colors. Sequence alignments of different miR391 target sites are shown.
